# Efficacy assessment of biocides or repellents for the control of *Sarcoptes scabiei* in the environment

**DOI:** 10.1186/s13071-015-1027-7

**Published:** 2015-08-12

**Authors:** Fang Fang, Charlotte Bernigaud, Kerdalidec Candy, Elise Melloul, Arezki Izri, Rémy Durand, Françoise Botterel, Olivier Chosidow, Weiyi Huang, Jacques Guillot

**Affiliations:** Parasitology Department, College of Animal Science and Technology, Guangxi University, Nanning, China; Research group Dynamyc, EnvA, UPEC, Maisons-Alfort & Créteil, France; Dermatology Department, Henri Mondor Hospital, AP-HP, UPEC, Créteil, France; Parasitology-Mycology Department, Avicenne Hospital, AP-HP, Bobigny, France

**Keywords:** *Sarcoptes scabiei*, Pyrethroids, Repellents, *in vitro* test

## Abstract

**Background:**

*Sarcoptes scabiei* infection is a contagious disease affecting both humans and animals. The transmission occurs either by direct contact or from the environment where mites could survive several days remaining infective. The number of products available for environmental control of *S. scabiei* is very limited. The objective of the present study was to assess the efficacy of biocides or repellents against *S. scabiei* var *suis*.

**Methods:**

Tested products included pyrethroids: permethrin, esdepallethrin and bioresmethrin, bifenthrin, cypermethrin and imiprothrin, cyfluthrin, tetramethrin and sumithrin. We also tested repellents: DEET, icaridin and IR3535. *Sarcoptes scabiei* var *suis* mites were collected from experimentally-infected pigs. For each test, 20 live mites of all motile stages were placed in a plastic Petri dish and sprayed uniformly by each product. Control mites were sprayed by distilled water. The study was performed in triplicate under room conditions and the mites were inspected under a stereomicroscope at intervals (5, 10, 15, 20, 25, 30, 40, 50, 60 min, 2, 3, 4, 5 and 24 h) after exposure to the products.

**Results:**

All the products, except the combination of tetramethrin and sumithrin (A-PAR®), were able to kill all mites within 24 h. The median survival time was 50 ± 30.4 min, 120 ± 309 min, 10 ± 5.9 min, 40 ± 36.8 min, 15 ± 7.3 min, 180 ± 417 min and 1440 ± 600 min when mites were exposed to permethrin 4 %, permethrin 0.6 %, esdepallethrin and bioresmethrin, bifenthrin, cypermethrin and imiprothrin, cyfluthrin, tetramethrin and sumithrin, respectively. The median survival time was 20 ± 6.5 min, 15 ± 4.3 min, 30 ± 42.1 min and 15 ± 4.9 min for DEET 25, DEET 50, icaridin 20 and IR3535 20 %, respectively.

**Conclusions:**

The results of the present study could support evidence-based use of biocides and repellents in households, hospitals and farms.

## Background

Scabies, or mange as it is called in animals, is a common ectoparasitic infection caused by the mite *Sarcoptes scabiei* [1]. This has been reported in 104 species across 27 families of domestic and wild animals [2]. Scabies is now recognized as an emerging/re-emerging infectious disease in humans, with an estimation of 100–300 million cases per year worldwide [3].

*Sarcoptes scabiei* burrows in the stratum corneum and stratum granulosum of the skin in both humans and animals [4, 5] and the transmission of scabies/mange acts through direct and indirect contact. Arlian *et al.* demonstrated that away from their hosts, mites are able to survive and remain infective for 24-36 h at 21 °C and 40-80 % relative humidity, and can even survive longer at lower temperatures with higher levels of humidity [6]. Generally, female and nymph mites survive longer than larvae and males in comparable conditions, with nymphs surviving up to 21 days at 10 °C and 97 % relative humidity [7].

Studies carried out on pigs and foxes showed that the transmission of *S. scabiei* occurred when uninfected animals were exposed to fomites [8, 9]. Mites were found from fomites in a survey in homes and nursing homes of scabies patients [10]. These factors coupled with the survival and infectivity of mites suggest that fomites could be a source of infection, especially in cases of crusted scabies which is characterized by the presence of thousands of mites [11–13]. On the other hand, Arlian *et al*. [11] showed that good environmental practices such as regular housekeeping, frequent bed linen changes and good hygiene practice could minimize fomite contamination.

In order to prevent possible infection from fomites, current protocols for treating scabies patients and mange animals include environmental measures such as by applying acaricidal spray, usually pyrethroids or pesticide [12, 14]. Surprisingly, no specific data on scabicidal effect is available, which hinders appropriate choices made by doctors and veterinarians. The main limitation has been the lack of a regular supply of adequate numbers of mites. The successful establishment of rabbit and pig models have made these tests possible [15–17].

So far, there have been plenty of commercially available products that can be used for environmental control of fleas, lice, ticks and mosquitoes. The objective of the present study is to assess the efficacy of biocides or repellents against *S. scabiei* var. *suis*. Tested products included pyrethroids and repellents.

## Methods

### *Sarcoptes* mites

*Sarcoptes* mites were collected from pigs maintained at CRBM (Centre de Recherche Bio Medicale), Maisons-Alfort, France. They were experimentally-infected as described by Mounsey [16]. All animals were maintained in strict accordance with good animal practices as defined by the French and European code of practice for the care and use of animals for scientific purposes (approval No. 02515.01). Inoculation was done by directly introducing mite-infected skin crusts deep into the ear canals of five-week old female piglets. Glucocorticoid treatment was initiated in naive piglets one week prior to inoculation and continued throughout the study period. With the pig model, first cutaneous lesions are visible two weeks after experimental infection and encrustment usually occurs after four weeks. The ear is the first place to develop lesions then lesions spread to the entire body of the pigs. The clinical score slowly increases after experimental infection and is stable after week 7. For the present study, mites were collected from the pigs in weeks 9 and 10. Crusts in the external ear canal were gently removed in a sterile Petri dish. Mites crawled out of the crusts in about half an hour. Then they were picked one by one with a needle and placed into a Petri dish.

### Products

The tested products were chosen from the products that were utilized for environmental control of mites, lice, fleas and other insects; and a repellent for mosquitoes, ticks and flies. These products are available in pharmacies, veterinary clinics or supermarkets in France. The components of biocides are mostly pyrethroids, some with one active compound, and others with more than one. Brands like Insect Ecran® and Pyréflor® have different targets and consequently include different compounds. All the products are sprays or aerosols. The list is shown in Table 1.

**Table 1:** Active compounds of the products tested in the present study

No.	Active compounds and concentration	Targets	Product names	Companies
1	IR3535 20 %	Repellent for lice	Paranix® 100 mL	Omega Pharma Barcelona, Spain
2	DEET 25 %	Repellent for mosquitoes, ticks & flies	Insect Ecran® 100 mL	Cooper, Melun, France
3	DEET 50 %	Repellent for mosquitoes, ticks & flies	Insect Ecran® 100 mL	Cooper, Melun, France
4	icaridin 20 %	Repellent of mosquitoes	Insect Ecran® 75 mL	Cooper, Melun, France
5	permethrin 4 %	Insecticide	Insect Ecran® 100 mL	Cooper, Melun, France
6	esdepallethrin 2.1 g/L, bioresmethrin 0.45 g/L	Environmental control of lice	Pyréflor® 150 mL	Clément-Thékan, France
7	bifenthrin 0.67 g/L	Environmental control of lice	Pyréflor® 150 mL	Ferlux, Cournond’Auvergne, France
8	cypermetrin 0.10 %, imiprothrin 0.10 %	Insecticide	Raid® 400 mL	S.C. Johnson, Mijdrecht, The Netherlands
9	permethrine 0.6 %, pyriproxyfen 0.05 %	Environmental control of fleas	Parastop® 500 mL	Virbac, Carros, France
10	cyfluthrin 0.16 g/L, pyriproxyfen 0.2 g/L	Environmental control of fleas	Advanthome® 250 mL	Bayer HealthCare Animal Health, Puteaux, France
11	tetramethrin 0.95 g/L, sumithrin 0.95 g/L	Disinfectant against *Sarcoptes* mites, lice, fleas & bedbugs	A-PAR® 200 mL	Omega Pharma, Châtillon, France

### Efficacy tests

Mites were tested within 3 h after they were harvested from the pigs. Live mites of all motile stages (*n* = 20) were placed in a plastic sterile Petri dish (3 cm in diameter). In each Petri dish, mites were sprayed uniformly until they were completely covered by the tested product. A control Petri dish was sprayed by distilled water. All Petri dishes were placed at room conditions (25 °C, 30-70 % relative humidity). The mites were examined under a stereomicroscope after 5, 10, 15, 20, 25, 30, 40, 50, 60 min, 2, 3, 4, 5, 24 h. Persistent immobility, even when stimulated with a needle was considered as death [18]. The study was performed in triplicate.

### Statistical analyses

The data were analyzed by Kaplan Meier survival curves; median survival times of scabies mites and significant differences between survival curves were calculated by a Log-rank test using JMP 11.0 software.

## Results

**Fig. 1:**
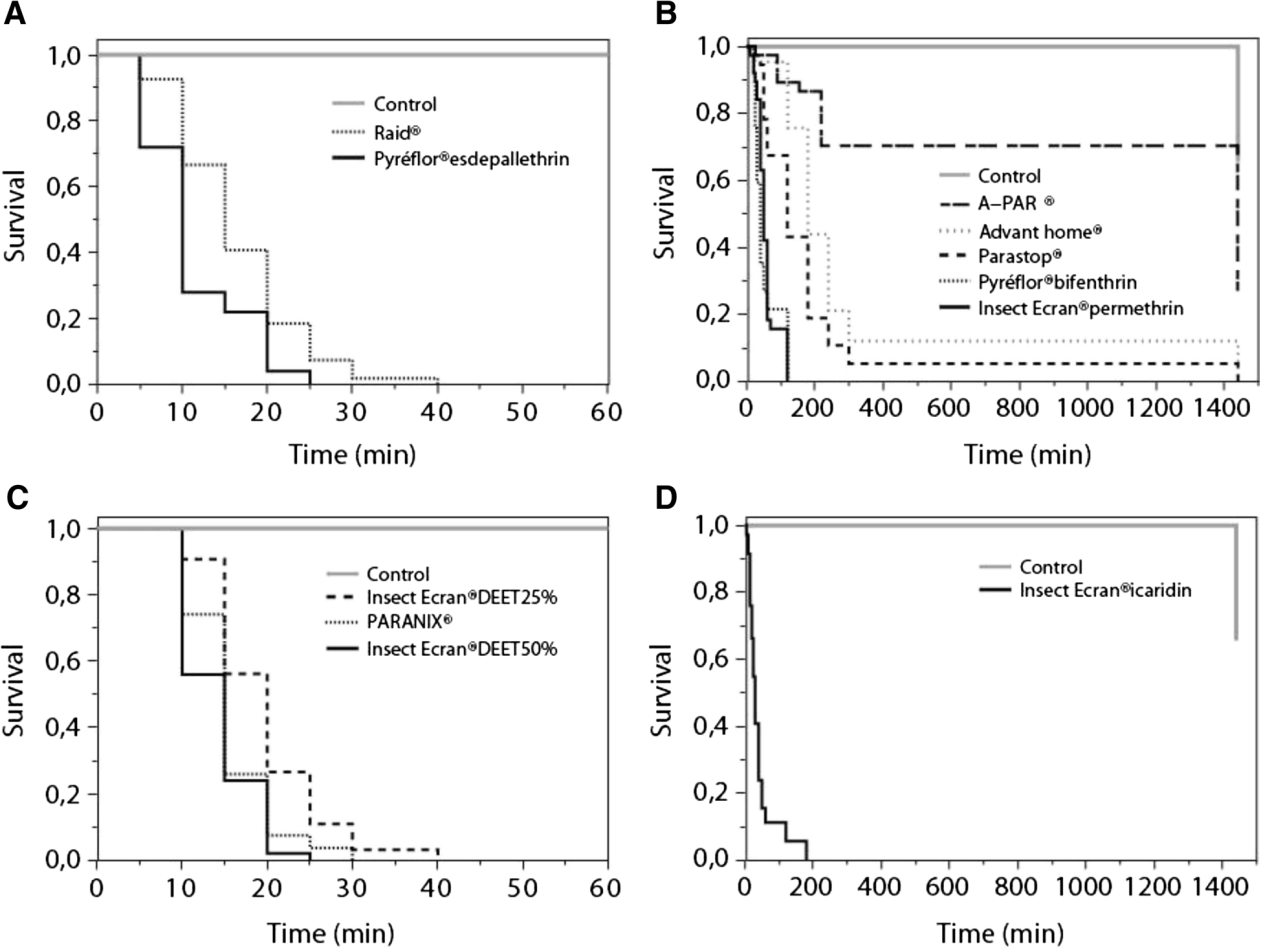
Survival curves of *Sarcoptes* mites exposed to biocides or repellents. **a**. Survival curves with fast-acting biocides (all the mites were killed within 40 min); **b**. Survival curves with other biocides. **c**. Survival curves with fast-acting repellents (all the mites were killed within 40 min); **d**. Survival curve with the other repellent, icaridin

With the *in vitro* test, we were able to demonstrate the scabicidal activity of several biocides and repellents. During the tests, we also observed that larvae and males were usually killed before females and nymphs. The survival curves for the different products are presented in Fig. 1. The median survival time of *Sarcoptes* mites varied from 10 to 1440 min (Table 2). Except A-PAR® (Table 1, product number 11), all the products kill all mites within 24 h (Fig. 1a and b). Statistically significant differences in mite survival time were observed for all tested products with the distilled water used as control. Although the main components of 7 products were pyrethroids, the variability in survival times of the mites was obvious. All mites were dead within 2 h when using Insect Ecran® (product No. 5), Pyréflor® (product No. 6), Pyréflor® (product No. 7) and Raid® (product No. 8). Of these, Pyréflor® (product No. 7) and Raid® (product No. 8), with a median survival time of 10 ± 5.87 min and 15 ± 7.31 min, showed a strong scabicidal effect. In contrast, approximately 5, 10 and 70 % of mites were still alive after 5 h sprayed with Parastop® (product No. 9), Advanthome® (product No. 10) and A-PAR® (product No. 11), respectively. A dose-dependent change in median survival time of permethrin-based products was observed, with the median survival time of 50 ± 30.4 min and 120 ± 309 min for permethrin 4 (product No. 5) and permethrin 0.6 % (product No. 9), respectively.

**Table 2:** Comparisons of Log-rank test pairwise survival time of *S. scabiei* var *suis* sprayed with different products in comparison with distilled water (negative control)

Product names	Median survival time (min)	Standard deviation	P value
Pyréflor® esdepallethrin	10	5.9	<.001
Insect Ecran® DEET 50 %	15	4.3	<.001
Paranix®	15	4.9	<.001
Raid®	15	7.3	<.001
Insect Ecran® DEET 25 %	20	6.5	<.001
Insect Ecran® icaridin	30	42.1	<.001
Pyréflor® bifenthrin	40	36.8	<.001
Insect Ecran® permethrin	50	30.4	<.001
Parastop®	120	309.0	<.001
Advanthome®	180	417.0	<.001
A-PAR®	1440	600.0	<.002
Distilled water	1440	-	-

The four repellent products were active against *S. scabiei* mites (Fig. 1c and d). DEET and IR3535 products killed all mites within 1 h, while icaridine took 3 h to kill all mites. DEET revealed small-scale dose-dependent scabicidal activity, the median survival times were 20 ± 6.5 min and 15 ± 4.3 min for 25 and 50 % of DEET, respectively.

## Discussion

There are several studies that used *in vitro* tests to assess the efficacy of current acaricides or essential oils that could be recommended for the treatment of scabies [18–21]. To the authors’ knowledge, the present study is the first one to assess products for the control of *S. scabiei* in the environment. The finding that larvae and males were killed before females and nymphs is in accordance with Arlian’s observation that female and nymph mites survived longer in comparable conditions [6]. Since we tested all motile stages of mites in the study, the varied number of females and nymphs could influence the result, especially for the products with low activity. Moreover, this can explain why the standard deviation values of some tested products were high. For the tests, we used distilled water as control, which is probably not always a good control to determine the specific action as solvents might also be involved. However, in biocides or repellents products used in the present study, the solvent was not mentioned by the manufacturers.

The active components of all the biocide products tested here belong to the pyrethroid family that is widely used in public health and agriculture throughout the world and generally considered to be the safest class of insecticides/acaricides available. They are known to work on voltage-dependent sodium channels in the nerve membrane [22]. The present study demonstrated that pyrethroids differed a lot regarding the speed of activity against *Sarcoptes* mites. Pyréflor® (product No. 6) was the most effective of all. It includes the active component esdepallethrin which is used for the treatment of human scabies in France [23]. Permethrin is not only used for the treatment of scabies, but is also recommended for environmental control of *S. scabiei*. In previous studies of *in vitro* tests against *S. scabiei*, the lethal time of 5 % permethrin was 480 min and 1320 min, respectively [19, 20], whereas it was 120 min with 4 % permethrin in the present study. This may be attributed to the method used for efficacy assessment of permethrin. In the current study permethrin was sprayed directly on the mites while Walton *et al*. [19, 20] applied 0.1 g of permethrin in a thin layer, using cotton swabs, on the bottom, top and sides of Petri dishes and then placed a mite into each Petri dish. Another explanation would be that the widespread use of permethrin in Australia since 1994 induced tolerance to this compound [24]. However, a previous study on the resistance marker gene *KDR* did not detect resistance to pyrethroids of scabies mites in France [23]. Compared to other biocide products, permethrin-based products were not so efficient in the present study. Raid® (product No. 8) is a common and cheap product used for household control of insects. Even though the concentration of its active components cypermetrin and imiprothrin was as low as 0.10 %, the efficacy on mites was notable. This result suggested that mites might be more susceptible to some pyrethroids. A-PAR® (product No. 11) is used in some French hospitals for the environmental control of *S. scabiei*. However, the present study showed that A-PAR® should not be recommended for this purpose.

Repellents are supposed to prevent arthropods from landing on the surface where they are applied (without a necessary killing effect). However, previous studies showed that DEET and IR3535 display insecticidal as well as acaricidal activity [25, 26]. In the present study, the repellents DEET, IR3535 and icaridine had acaricidal activity and differed in their effects on mites. Although the exact mode of action and molecular target of repellents remain controversial, there is evidence that repellents exert their effects through interactions with odorant receptors and gustatory receptors in insects [27]. It was also demonstrated that DEET induced a neurotoxic effect on insects by disrupting the calcium equilibrium in the nerve cells [28]. Faulde *et al.* showed that Both DEET and IR3535 revealed a dose-dependent insecticidal as well as acaricidal activity, and DEET exhibited a higher knockdown effect and mortality than IR3535 [26]. From the present study, DEET also showed a small-scale dose-dependent scabicidal activity, but IR3535 may work better than DEET against *S. scabiei*. Although the repellent products, especially DEET and IR3535, caused relatively high scabicidal effect, one cannot infer that repellents work better than pyrethroids.

As commercial products are complex in their composition and varied in concentration, further investigation is necessary to determine the scabicidal effect of individual chemicals.

## Conclusions

The present study demonstrated that two biocides: Pyréflor® (product No. 6) and Raid® (product No. 8) and three repellents: Paranix® (product No. 1), Insect Ecran® (product No. 2) and Insect Ecran® (product No. 3) were able to kill *S. scabiei* within 40 min when they were sprayed on the mites. On the contrary, A-PAR® (product No. 11) did not prove to be a good choice for environmental control of *Sarcoptes* mites. The results of the present study might support evidence-based use of biocides and repellents for households, hospitals and farms but should be repeated as insecticide pressure and subsequent resistance may change results over time.

## References

[1] Chosidow O (2006). Scabies. N Engl J Med.

[2] Currier RW, Walton SF, Currie BJ (2011). Scabies in animals and humans: history, evolutionary perspectives, and modern clinical management: Currier et al. Ann N Y Acad Sci.

[3] Fuller LC (2013). Epidemiology of scabies. Curr Opin Infect Dis.

[4] McCarthy JS (2004). Scabies: more than just an irritation. Postgrad Med J.

[5] Hengge UR, Currie BJ, Jäger G, Lupi O, Schwartz RA (2006). Scabies: a ubiquitous neglected skin disease. Lancet Infect Dis.

[6] Arlian LG (1989). Biology, host relations, and epidemiology of *Sarcoptes scabiei*. Annu Rev Entomol.

[7] Arlian LG, Vyszenski-Moher DL, Pole MJ (1989). Survival of adults and developmental stages of *Sarcoptes scabiei* var. *canis* when off the host. Exp Appl Acarol.

[8] Smith HJ (1986). Transmission of *Sarcoptes scabiei* in swine by fomites. Can Vet J Rev Vét Can.

[9] Samuel WM, Kocan AA, Pybus MJ, Davis JW (2001). Parasitic Diseases of Wild Mammals.

[10] Arlian LG, Estes SA, Vyszenski-Moher DL (1988). Prevalence of Sarcoptes scabiei in the homes and nursing homes of scabietic patients. J Am Acad Dermatol.

[11] Chosidow O (2000). Scabies and pediculosis. Lancet.

[12] Walton SF, McBroom J, Mathews JD, Kemp DJ, Currie BJ (1999). Crusted scabies: a molecular analysis of *Sarcoptes scabiei* variety *hominis* populations from patients with repeated infestations. Clin Infect Dis.

[13] Thomas MC, Giedinghagen DH, Hoff GL (1987). Brief report: an outbreak of scabies among employees in a hospital-associated commercial laundry. Infect Control.

[14] Curtis CF (2004). Current trends in the treatment of *Sarcoptes*, *Cheyletiella* and *Otodectes* mite infestations in dogs and cats. Vet Dermatol.

[15] Arlian LG, Runyan RA, Estes SA (1984). Cross infestivity of *Sarcoptes scabiei*. J Am Acad Dermatol.

[16] Mounsey K, Ho M-F, Kelly A, Willis C, Pasay C, Kemp DJ, McCarthy JS, Fischer K (2010). A tractable experimental model for study of human and animal scabies. PLoS Negl Trop Dis.

[17] Casais R, Dalton KP, Millán J, Balseiro A, Oleaga Á, Solano P, Goyache F, Prieto JM, Parra F (2014). Primary and secondary experimental infestation of rabbits (*Oryctolagus cuniculus*) with *Sarcoptes scabiei* from a wild rabbit: factors determining resistance to reinfestation. Vet Parasitol.

[18] Pasay C, Mounsey K, Stevenson G, Davis R, Arlian L, Morgan M, Vyszenski-Moher D, Andrews K, McCarthy J (2010). Acaricidal activity of eugenol based compounds against scabies mites. PLoS One.

[19] Walton SF, Myerscough MR, Currie BJ (2000). Studies in vitro on the relative efficacy of current acaricides for *Sarcoptes scabiei* var. *hominis*. Trans R Soc Trop Med Hyg.

[20] Walton SF, McKinnon M, Pizzutto S, Dougall A, Williams E, Currie BJ (2004). Acaricidal activity of *Melaleuca alternifolia* (tea tree) oil: *in vitro* sensitivity of *Sarcoptes scabiei* var *hominis* to terpinen-4-ol. Arch Dermatol.

[21] Du Y-H, Jia R-Y, Yin Z-Q, Pu Z-H, Chen J, Yang F, Zhang Y-Q, Lu Y (2008). Acaricidal activity of extracts of neem (*Azadirachta indica*) oil against the larvae of the rabbit mite *Sarcoptes scabiei* var. *cuniculi in vitro*. Vet Parasitol.

[22] Vijverberg HP, vanden Bercken J (1990). Neurotoxicological effects and the mode of action of pyrethroid insecticides. CRC Crit Rev Toxicol.

[23] Andriantsoanirina V, Izri A, Botterel F, Foulet F, Chosidow O, Durand R (2014). Molecular survey of knockdown resistance to pyrethroids in human scabies mites. Clin Microbiol Infect.

[24] Mounsey KE, Holt DC, McCarthy J, Currie BJ, Walton SF (2008). Scabies: molecular perspectives and therapeutic implications in the face of emerging drug resistance. Future Microbiol.

[25] Licciardi S, Herve JP, Darriet F, Hougard J-M, Corbel V (2006). Lethal and behavioural effects of three synthetic repellents (DEET, IR3535 and KBR 3023) on *Aedes aegypti* mosquitoes in laboratory assays. Med Vet Entomol.

[26] Faulde MK, Albiez G, Nehring O (2010). Insecticidal, acaricidal and repellent effects of DEET- and IR3535-impregnated bed nets using a novel long-lasting polymer-coating technique. Parasitol Res.

[27] Dickens JC, Bohbot JD (2013). Mini review: mode of action of mosquito repellents. Pestic Biochem Physiol.

[28] Lapied B, Pennetier C, Stankiewicz M, Gautier H, Fournier D, Hougard JM, Corbel V (2006). The insect repellent DEET exerts neurotoxic effects through alterations of both neuronal function and synaptic transmission.

